# Modeling the Ruminant Placenta-Pathogen Interactions in Apicomplexan Parasites: Current and Future Perspectives

**DOI:** 10.3389/fvets.2020.634458

**Published:** 2021-01-21

**Authors:** Iván Pastor-Fernández, Esther Collantes-Fernández, Laura Jiménez-Pelayo, Luis Miguel Ortega-Mora, Pilar Horcajo

**Affiliations:** Animal Health and Zoonoses (SALUVET) Group, Animal Health Department, Faculty of Veterinary Sciences, Complutense University of Madrid, Madrid, Spain

**Keywords:** ruminants, placenta, neosporosis, toxoplasmosis, *in vitro* models, *in vivo* models, *ex vivo* models

## Abstract

*Neospora caninum* and *Toxoplasma gondii* are one of the main concerns of the livestock sector as they cause important economic losses in ruminants due to the reproductive failure. It is well-known that the interaction of these parasites with the placenta determines the course of infection, leading to fetal death or parasite transmission to the offspring. However, to advance the development of effective vaccines and treatments, there are still important gaps on knowledge on the placental host-parasite interactions that need to be addressed. Ruminant animal models are still an indispensable tool for providing a global view of the pathogenesis, lesions, and immune responses, but their utilization embraces important economic and ethics restrictions. Alternative *in vitro* systems based on caruncular and trophoblast cells, the key cellular components of placentomes, have emerged in the last years, but their use can only offer a partial view of the processes triggered after infection as they cannot mimic the complex placental architecture and neglect the activity of resident immune cells. These drawbacks could be solved using placental explants, broadly employed in human medicine, and able to preserve its cellular architecture and function. Despite the availability of such materials is constrained by their short shelf-life, the development of adequate cryopreservation protocols could expand their use for research purposes. Herein, we review and discuss existing (and potential) *in vivo, in vitro*, and *ex vivo* ruminant placental models that have proven useful to unravel the pathogenic mechanisms and the host immune responses responsible for fetal death (or protection) caused by neosporosis and toxoplasmosis.

## The Ruminant Placenta: a Battlefield Between Host and Apicomplexan Parasites

The placenta plays a critical role in the pathogenesis of neosporosis and toxoplasmosis as it acts as a biological barrier that must be crossed by the parasite to reach the fetus. In addition, from an immunological point of view, it is a unique organ. On one hand, it facilitates embryo implantation promoting immunological tolerance to the conceptus. On the other hand, it mobilizes a number of defense mechanisms against pathogens potentially harmful to the developing fetus (1). Despite this, *Neospora caninum* and *Toxoplasma gondii*-the etiologic agents of bovine neosporosis and sheep (and goat) toxoplasmosis, respectively, are still able to replicate in the placental tissues, compromising pregnancy success. This outcome is triggered by two different mechanisms related to parasite replication itself and the maternal immune response developed against the replicating parasites, both potentially harmful for the placenta (2). In this review, we aim to summarize available and potential models to study effects of infection by these parasites at the maternal-fetal interface, thus encouraging future research focused on expanding the understanding of the pathogenesis of bovine neosporosis and ovine toxoplasmosis.

### Structure and Function of the Placenta in Ruminants

**Figure 1:**
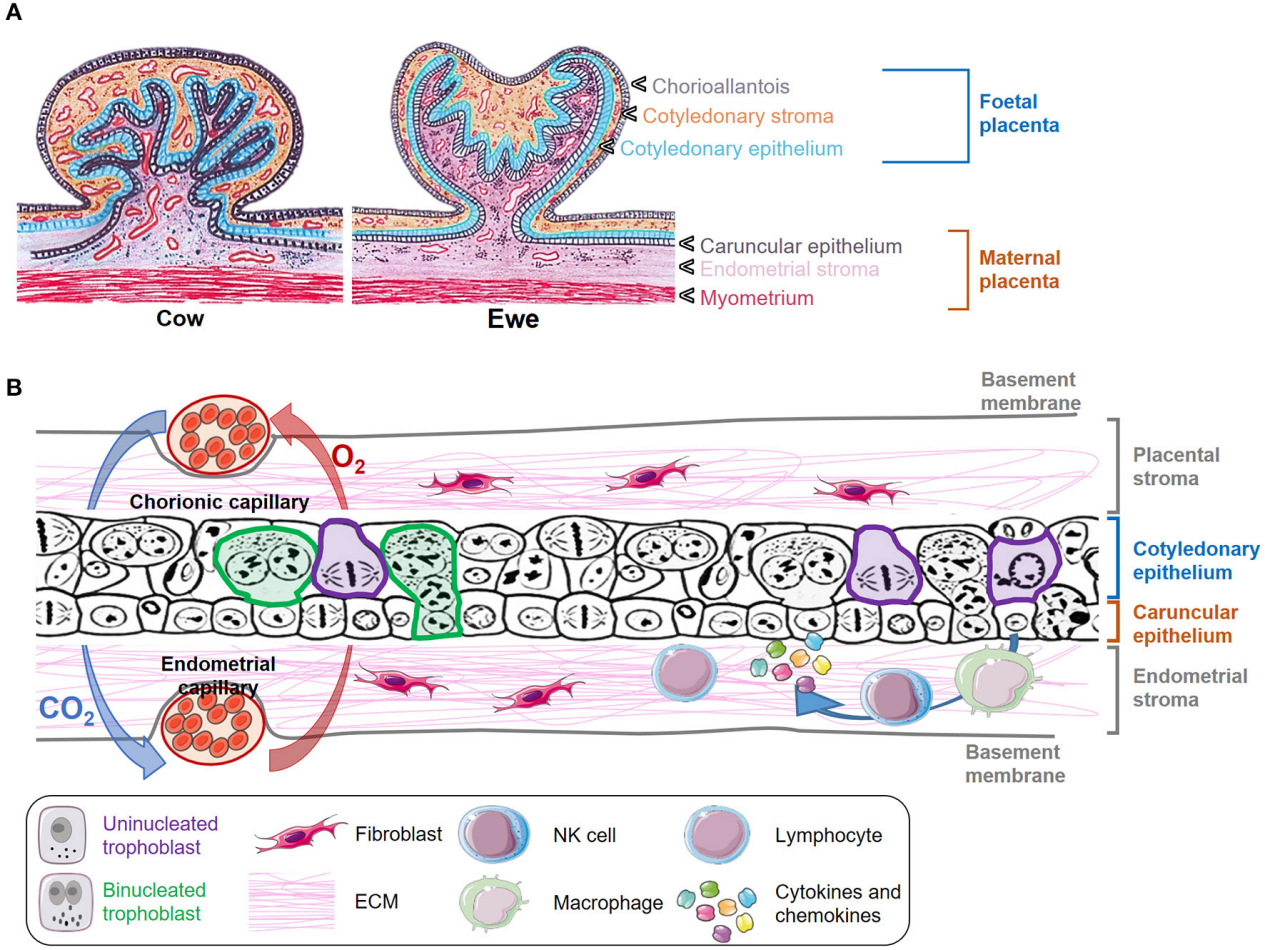
Morphology and structure of the placenta in ruminants. **(A)** Transverse section of a cow and ewe placentome indicating the different layers of the fetal and maternal placenta. **(B)** Schematic representation of the microscopic structure of placentomes. Some uninucleated trophoblast cells are delineated in purple, and some binucleated trophoblast cells are marked in green. Gas interchange between chorionic and endometrial capillary is represented with blue and red arrows. The presence of fibroblasts and immune cells is also depicted.

**Figure 2:**
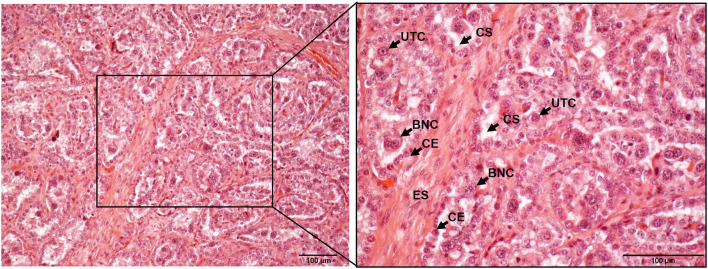
Histological composition of bovine placentome samples stained with hematoxylin and eosin and observed under the microscope at 10x (left) and 20x (right). ES, endometrium stroma; CE, caruncular epithelium; UTC, uninucleated trophoblast cell; BNC, bininucleated trophoblast cell; CS, cotyledonary stroma. Scale-bars: 100 μm.

In mammals, the growth and survival of the fetus depend on the placenta, a temporary organ formed by an intricate of maternal and fetal tissues that keep both individuals connected. The ontogeny and morphology of the ruminant placenta have been thoroughly reviewed in the last years (3–5), and thus we will focus this review on the key aspects of placental structure necessary to understand how host-pathogen interactions occur. Domestic ruminants present chorioallantoic, villous, and cotyledonary placentae. These terms refer to the fusion of the chorion and allantois in discrete areas of the fetal placenta to form villous projections named cotyledons. Considering the number of layers that act as a barrier between the fetal and maternal circulation in these species, placentae can also be classified as synepitheliochorial due to the replacement of the maternal epithelial layer opposed to the fetal chorion by a foeto-maternal syncytial layer (6) (Figures 1A, 2). In ruminants, the implantation process begins at 16 (sheep) or 20 (cow) days post coitum, when the chorionic epithelium of the embryo (formed by trophoblast cells) develops folds which fit into raised projections of the uterine wall (caruncles). These chorionic folds form villous projections (chorioallantoic villi) that branch and extend toward the caruncular wall with which interdigitates forming septa. In turn, the caruncular septa extend branching projections in the opposite direction, thus forming a network of fetal villi apposed within maternal crypts. The combination of a fetal cotyledon and a maternal caruncle is termed placentome, which is considered the functional units where the main placental functions take place (Figures 1A, 2) (3–6).

Trophoblast cells are key components of the placenta, where two different populations have been described: the uninucleated trophoblast cells, which form the majority of the fetal interface and are primarily involved in nutrient exchange, and the binucleate cells (BNC), which fuse with caruncular syncytial cells and are involved in the synthesis of hormones such as placental lactogen and progesterone (7, 8) (Figures 1B, 2). The extracellular matrix (ECM) is another key component of placenta that mediates trophoblast attachment to the uterine endometrium, induces cell differentiation (9), acts as a reservoir of growth factors (10), and serves as a track on which cells can migrate along (11). ECM consists of a mixture of proteoglycans, glycosaminoglycans, and structural components such as different types of collagen, fibronectin, and laminin that are mainly synthesized by fibroblasts. These cells also produce matrix metalloproteinases (MMP), such as collagenases, gelatinases and matrilysins that degrade the ECM components. The activity of degrading enzymes is highly regulated by tissue inhibitors of MMP (TIMPs) and chemokines, cytokines, and growth factors, but the imbalance of these proteins has been associated to different pathologic conditions and with the dissemination of parasites and infected cells through the placental barrier (12, 13). Both pregnancy and delivery require a constant placenta remodeling that involves a delicate balance between ECM synthesis and degradation (4).

Regarding its functions, the placenta forms an interface between the maternal and fetal circulation, thus acting as the digestive, respiratory, excretion, metabolic and endocrine system of the fetus. Low molecular weight substances (water, electrolytes, urea, uric acid, creatine, oxygen, CO_2_, and some drugs) can cross the placenta by simple diffusion, other metabolites (glucose, amino acids, fatty acids) associate to specific carriers to pass the placenta, and more complex molecules (proteins, phospholipids, neutral fats) are modified to other simpler compounds before crossing the placental membrane, and then resynthesized. Gas interchange between the dam and the fetus is favored by the higher oxygen concentration and the partial CO_2_ pressure in the maternal circulation, but also by the higher affinity of fetal hemoglobin for oxygen (14). The placenta also secretes the hormones necessary for pregnancy maintenance, adaptation of the maternal metabolism, fetal growth, parturition and even lactation. However, the endocrine gestational profiles differ between domestic ruminant species (15).

In addition, the maternal-fetal interface constitutes a physical barrier that protects the fetus against germs and drugs that may circulate in the maternal blood stream. However, some pathogens are able to cross this barrier and infect the fetus due to their reduced size (e.g., viruses), or their ability to replicate both in maternal and fetal placental cells (e.g., *T. gondii, N. caninum, Brucella abortus, Chlamydia abortus*, among others), thus resulting in tissue damage and potential risk of abortion. Pregnancy success depends on a precise immune modulation able to protect the fetus from infectious agents, but also capable of providing a receptive environment for the development of a semi-allogenic conceptus. In this sense, resident immune cells, as well as trophoblast and caruncular cells, can respond to these infections by secreting cytokines and chemokines that will ultimately recruit immune cells in the damaged area (16–18). The local immune modulation that takes place during pregnancy is a complex process which has not been fully understood yet. It is known that gestation drives immune responses toward tolerance and secretion of cytokines that promote placental growth and function. This shift is characterized by reduced expression of cytokines associated with inflammation (Th1/Th17) and increased expression of cytokines that suppress inflammation (Th2) (19). Nonetheless, it has been shown that the displacement of the cytokine equilibrium toward Th1 responses under certain pathological conditions leads to an immunomediated abortion (20).

### *Neospora caninum* and *Toxoplasma gondii* as Relevant Causes of Reproductive Failure in Domestic Ruminants

Neosporosis and toxoplasmosis are cosmopolitan diseases leading to reproductive failure and important economic losses in farm ruminants (21, 22). In addition, toxoplasmosis is recognized as a worldwide relevant parasitic zoonosis (23). Both diseases are caused by closely related cyst-forming apicomplexan parasites, *N. caninum* and *T. gondii*, respectively, that share many morphological and biological features (24, 25).

Neosporosis greater relevance lies in cattle, as the disease causes severe economic losses in dairy and beef industries. Abortion is the main clinical sign of bovine neosporosis. Fetuses in *Neospora*-infected dams may die *in utero* and be reabsorbed, mummified, autolysed or stillborn. Cows of any age may abort from 3 months of gestation to term, with most abortions observed at 5–7 months of gestation (21), and transplacental infections after mid-pregnancy may produce calves born alive but with clinical signs or clinically normal but persistently infected. Reproductive failure can sporadically take place in other ruminant intermediate hosts including sheep, goats, and deer (21, 26, 27). Currently, there are no effective vaccines or treatments against infection by *N. caninum* (28, 29) and control options are based on diagnosis, biosecurity measures and management practices (21).

Toxoplasmosis is considered a major cause of reproductive losses in sheep and goats worldwide (22, 30). Infection during pregnancy usually results in fetal death (early-gestation); stillborn or the birth of a weak lamb, sometimes accompanied by a small, mummified fetus (mid-gestation); or the birth of healthy lambs, but congenitally infected (late-gestation) (31). Toxoplasmosis has been recognized as responsible of 10–23% of ovine abortions in Europe or USA (32) and could be responsible for between 680,000 and 1,360,000 abortions annually in the European Union. In addition, small ruminants may play a major role in its transmission to humans (33). The control of ovine toxoplasmosis is primarily based on preventing its horizontal transmission via oocysts present in cat feces and on the establishment of a vaccination program with a live attenuated S48 strain (Toxovax™, MSD).

#### Parasite Biology

*Neospora* and *Toxoplasma* share many features in their facultative heteroxenous coccidian life cycles, including three invasive stages: (i) the rapidly replicating tachyzoites, (ii) the slowly replicating or quiescent bradyzoites harbored in tissue cysts present in brain and skeletal muscle, and (iii) the sporulated oocysts containing sporozoites. However, both parasites differ in their host range, the zoonotic potential (demonstrated only for *T. gondii*), and the relevance of horizontal and vertical routes of transmission in maintaining the infections in the nature (21, 34). In both cases, sexual multiplication takes place exclusively in the intestine of the definitive host, while asexual multiplication occurs in different tissues of the intermediate hosts. Infection of intermediate hosts occurs by ingestion of food or water contaminated with oocysts or tissues containing tissue cysts. Upon ingestion, sporozoites or bradyzoites are released in the small intestine and invade intestinal epithelial cells, where they transform into tachyzoites, responsible for clinical signs during the acute phase of the disease. Both parasites establish its niche inside the host cytosol by forming a parasitophorous vacuole, inside which it replicates. The lytic cycle of both parasites is a tightly regulated process which includes adhesion to the host cell, invasion, parasitophorous vacuole formation, multiplication, and egress. The succession of cycles permits the parasite to proliferate and disseminate intraorganically during the acute phase of infection and is responsible for the lesions present in the host, being a consequence of cell lysis and the immune reaction triggered locally. In fact, it is thought that this host immune pressure is required by the parasite to convert into bradyzoites and form orally infectious tissue cysts, thus favoring further transmission (21, 34).

*Neospora caninum* life cycle involves canids as definitive hosts (dogs, gray wolves, coyotes, and dingoes), and cattle, other ungulates and dogs as intermediate hosts. While parasite DNA and/or clinical disease have been detected in a wide range of domestic and wild animals, dogs and cattle are the most relevant hosts and maintain the domestic life cycle of the parasite (21). Despite serological evidence of parasite exposure, *N. caninum* is not considered to be zoonotic as presence of the parasite has not been detected in human tissues (35). Postnatal infection by ingestion of oocysts (horizontal transmission) and transplacental passage from the dam to the fetus (vertical transmission) are the only demonstrated transmission routes for cattle and other intermediate hosts. Two different forms of transplacental transmission have been described in bovine neosporosis. Reactivation of bradyzoites in persistently infected dams during gestation is responsible for endogenous transplacental transmission, considered the main transmission route in cattle. Less frequently, exogenous transplacental transmission occurs when dams are primarily infected during gestation by ingestion of oocysts. Both exogenous and endogenous fetal infection may result in abortion or stillbirth, or in most cases, in the birth of clinically healthy calves, but congenitally infected. The ingestion of bradyzoites contained in tissue cyst from infected tissues of the intermediate host is the most likely source of infection for the definitive host (21).

Domestic cats and other felids are definitive hosts of *T. gondii*, and only they can shed environmentally resistant oocysts with the feces upon infection. After one to few days of environmental maturation (sporulation), oocysts become infective to a large variety of warm-blooded intermediate hosts, including livestock, synanthropic species, wild animals, and humans (36). The most important route of transmission of *T. gondii* to small ruminants is the horizontal, occurring after oral uptake of sporulated oocysts contaminating fodder or water (36). It is generally assumed that only 2% of sheep become infected congenitally and 4% of the persistently infected sheep transmit the infection to their offspring (37). Endogenous transplacental transmission of the parasite has also been described in goats (38). Recent descriptions from Brazil seem to corroborate the relevance of this route of transmission associated to certain breeds or *T. gondii* strains or genotypes (39, 40).

#### Factors Influencing Abortion and Transmission

The pathogenesis of bovine neosporosis and ovine toxoplasmosis is complex and not yet fully understood. Both *N. caninum* and *T. gondii* tachyzoites replicate and disseminate throughout the host tissues, including the placenta. Parasites seem to first reach and infect the caruncular epithelium, then spread to the trophoblast cells within the cotyledons, and eventually reach and replicate in the fetal tissues (41). Tissue damage and altered placental functions due to parasite replication have been proposed as mechanisms triggering the abortion (2). However, considering the role of the placenta as an immune-modulating organ and the ability of the parasite replication to alter its immunological balance, an immune-mediated mechanism has been also proposed as a possible cause of abortion (42, 43). Nevertheless, clinical outcomes of both diseases are also strongly associated with the period of gestation at which primary infection occurs (44, 45). This is because the response generated against the parasites is highly dependent not only on the status of the dam's immune system, but also the immunological maturity of the fetus (41, 46, 47). In addition, susceptibility to the disease is heavily determined by the parasite strain. In *N. caninum* most of the isolates obtained to date have been classified as low-, moderate- and high-virulence isolates based on their *in vivo* (mouse models) and *in vitro* (non-bovine cell lines) behavior (48–51). These isolates have shown a similar behavior in pregnant bovine models, demonstrating clear variations in their ability to produce abortion and vertical transmission after experimental infections (46, 52, 53). In *T. gondii*, virulence is defined by the number of tachyzoites that are needed to cause mortality in the 50% of mice inoculated with the parasite (LD50). In the house mouse (*Mus musculus domesticus*) type I strains are very virulent (LD100 of 1 parasite), whereas type II and III strains display milder virulence (LD50 of 103 and 105 parasites, respectively) (54). However, little is known about the correlation of *Toxoplasma* strain virulence in mice with other animal species, including humans (55).

## Models for the Study of Host-Pathogen Interactions at the Maternal-Fetal Interface

The use of animal models secures a unique opportunity to obtain a biological snapshot of the effects of infection with *N. caninum* or *T. gondii* during pregnancy, and thus, an excellent approach to analyze the interactions between the ruminant placenta and such parasites. Several pregnant cattle and sheep models have been employed so far, providing a powerful tool for the researchers working on this field. These models pose a number of disadvantages, including higher costs, longer experimental periods and reduced numbers of variables to be studied, not to mention the need to comply with the 3R principle. Consequently, different research groups have developed alternative simplified cell culture models based on the two major populations of the mature placenta: trophoblast and caruncular cells. These systems have provided useful information on the behavior of specific cell subsets when challenged with *N. caninum* or *T. gondii*, but offering a partial vision of the cellular processes that take place in the placenta, as they fail to mimic its complex structure (16, 56, 57). The use of *in vitro* models is mainly constrained by the lack of stimulation and activity of the immune cells resident or attracted to the placenta, the omission of host's ability to compensate for stress conditions, and the absence of the ECM, which is not only responsible for placental remodeling and function, but is also involved in the pathogenesis of some diseases, including toxoplasmosis (16, 58, 59). In this sense, and considering that explant cultures are able to retain the placental architecture and the dynamic relationship between fetal and maternal tissues, the use of *ex vivo* approaches seems an appealing alternative to deepen in the study of host-parasite relationships as they offer a mid-point between *in vivo* and classical *in vitro* systems (60).

### *In vivo* Ruminant Models

The use of animal models has played an integral part of our current understanding of pathogenesis of neosporosis and toxoplasmosis, since many features of pregnancy such as the development of the uteroplacental circulation, fetal growth velocity and development, and local immune responses have no *in vitro* counterpart. In this section we summarize the *in vivo* bovine and ovine models used to study the interaction of *N. caninum* and *T. gondii*, respectively, with the placenta, both in natural and experimental animal models, and discuss their benefits and difficulties.

#### Natural Infection Models

Collection of placental samples from naturally infected animals would be the ideal approach to study host-parasite interactions at the maternal-fetal interface, especially at the early stages of the description of an unknown disease. However, many variables cannot be controlled in such studies, including parasite isolate and challenge dose, obstructing their interpretation.

##### Bovine Neosporosis

Natural infections are the only model available to study endogenous transplacental transmission of *N. caninum*. In naturally infected heifers, the detection of an increase in the levels of specific antibodies has been related to a reactivation on the chronic infection during gestation (61, 62). Following these findings, Rosbottom et al. (63) monitored monthly fluctuations of *N. caninum*-antibodies during gestation in naturally infected cows, which were euthanized when recrudescence was detected (mid-gestation) and used to study the immune response, pathological changes and parasite loads in the placenta (63). This study showed that all the fetuses were alive, but parasite had already invaded the placenta and reached the fetal tissues when recrudescence was detected. Necrosis in the fetal villi and CD4+ and CD8+ T cells infiltration was also observed, with an increase of expression of pro-inflammatory (IFN-γ, IL-12, and TNF-α) and regulatory (IL-4 and IL-10) cytokines. Authors stated that the response in the placenta was not polarized toward either a Th1 or a Th2 phenotype, which may have an important role in controlling parasite multiplication.

##### Ovine Toxoplasmosis

Unlike neosporosis, the horizontal transmission is the main route that *T. gondii* exploits to infect small ruminants in the environment. Previous works carried out with naturally infected animals focused on the study of the consequences of the infection, with little information about the interactions of the parasite with the placenta. All these studies described the presence of multifocal areas of necrosis, commonly associated with the infiltration of non-purulent lymphoid in placentomes when abortion occurred (34).

#### Experimental Infection Models

Most of our current knowledge of parasite-placental interactions is derived from experimental infections in animals, and fortunately these can be made in the main target species: cattle for bovine neosporosis and sheep for ovine toxoplasmosis. Despite the use of rodent-based models has been extensively reported for *N. caninum*, differences in the type of placentation and length of gestation prevent direct extrapolations to ruminants. Marked differences between the ovine and murine models of toxoplasmosis have been also demonstrated, both in its clinical presentation and in the immune mechanisms to control infection (64). In mice the activation of TLR-11 and TLR-12 seem essential to control the infection, but these genes do not exist in sheep, cattle and humans (65, 66). In addition, NK cells are regarded as one of the key players of the placental innate immune response of mice against *T. gondii* (67), but they are notably absent from the ovine placenta during normal pregnancy (68) and ovine toxoplasmosis (69). Furthermore, and contrary to what has been described in mice (70), infection with *N. caninum* does not cause a clear predominance of Th1 or Th2 responses in ruminants (63, 71, 72). All these differences between mice and ruminants could reflect that the first are not natural hosts for *N. caninum* as suggested earlier (73), thus highlighting the relevance of using ruminant models for the study of these diseases.

**Figure 3:**
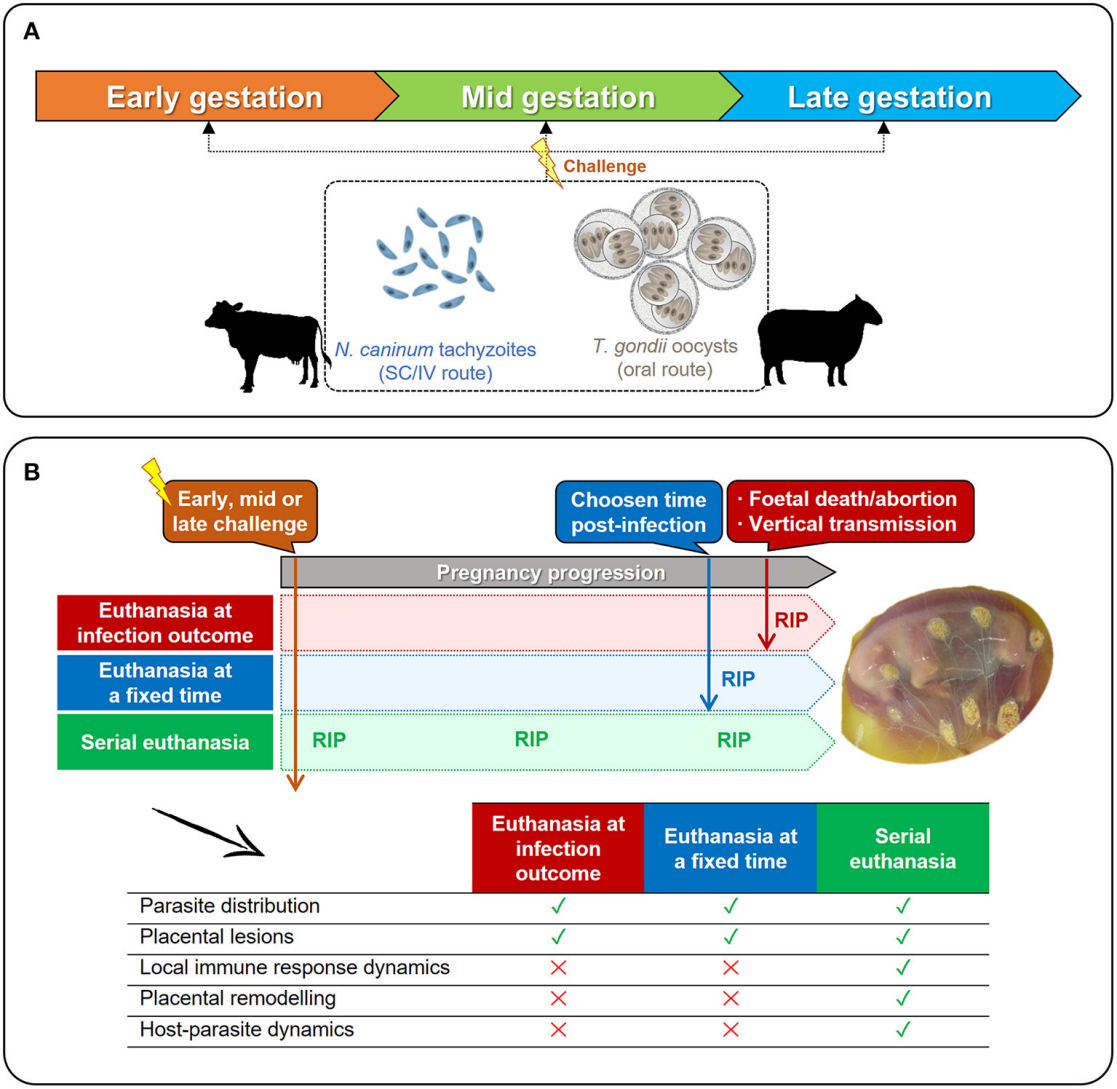
Available *in vivo* models to study the interactions between *N. caninum* or *T. gondii* and the ruminant placenta. **(A)** Pregnant cows or ewes can be experimentally infected at any term of gestation with *N. caninum* tachyzoites (subcutaneous –SC– or intravenous –IV– administration) or with sporulated oocysts of *T. gondii* (oral administration). **(B)** Regardless the time of challenge (early-, mid-, or late-gestation), pregnant females can be euthanized when clinical signs are detected (fetal death, abortion) or right after delivery; or can be serially euthanized after infection, before the appearance of clinical signs. As shown in the table, the time of euthanasia (RIP) will determine which parameters can be studied, and which cannot.

Knowledge on the available models is a prerequisite to reach appropriate conclusions, as interpretation of experimental findings varies between them. The Figure 3 summarizes the main ruminant models and experimental designs used to study parasite interactions with the placenta for both diseases. In all of them, experimental infection is achieved by subcutaneous or intravenous inoculation of *in vitro*-cultured tachyzoites (*N. caninum*) or by oral administration of sporulated oocyst (*T. gondii*) at the three terms of gestation (Figure 3A). Depiste models of oral and parental challenge have been also described for *N. caninum* and *T. gondii*, respectively, these have not been considered in this review because interactions with the placenta were scarcely analyzed and/or do not represent the main routes of transmission under natural conditions (44, 74, 75). Regardless the time and parasite used for infection, three main models can be defined (Figure 3B). Firstly, placental samples can be collected at the infection outcome, either when fetal death is detected *in utero* by ultrasound, or when abortion occurs. However, samples collected from abortions are often degraded with high degree of autolysis and/or display cellular and vascular changes related to placental expulsion rather than the infection. Secondly, placental samples can be obtained at a fixed time after infection, independently of the occurrence of fetal death of abortion. Lastly, a serial euthanasia at different times post-infection can be performed, allowing a thorough study of the infection dynamics, but preventing to track the progression of the parasite within the same host, which may yield relevant data. Surgical approaches such as the carunculectomy could be used to track single animals along the time, but in return, the integrity of the placental barrier could result compromised, affecting the reliability of the results.

It is worth to mention that the success of these experimental models also relies on the selection of appropriate animals. Although high individual variations are expected, uniform animals in terms of breed, weight and age should be selected. In addition, animals should be preferably primiparous and free from the main reproductive infectious pathogen agents: *N. caninum, Brucella abortus*, bovine viral diarrhea virus, *Leptospira interrogans* serovar Hardjo and infectious bovine rhinotracheitis virus for cattle; and *N. caninum, T. gondii*, border disease virus, Schmallenberg virus, *Brucella* spp., *Coxiella burnetii*, and *C. abortus* for sheep. Moreover, selected animals could be vaccinated against the main abortifacient agents, and then submitted to estrus synchronization and artificial insemination or natural mating. Once pregnant, females should be housed in dog- and cat- proof facilities and handled with care until implantation occurs. At the beginning of pregnancy, pregnant females require specific handling and feeding necessities and tolerate stress worse than non-pregnant ones, which can affect negatively on the implantation process. All these requirements involve higher costs, as experienced staff and large facilities are needed, implying that only few institutions can carry out such experiments.

With regard to neosporosis, a comprehensive review of the ruminant models used for neosporosis was published in 2014 (46). This paper highlighted the need of developing a normalized model of exogenous transplacental transmission (isolates, inoculation route, inoculum size, ruminant species, breed, infection times at pregnancy, sample collection timelines) to allow comparisons between studies performed by different research groups. In the last years, we have focused our efforts on the normalization of some variables including parasite isolate, challenge dose, and route of inoculation. The Nc-Liverpool, Nc-1, Nc-BPA1, Nc-Illinois, and Nc-6 Argentina isolate have been traditionally employed to perform experimental infections, showing a broad range of clinical outcomes. However, none of these isolates have been either extensively characterized or maintained in a low number of passages to preserve their traits. For this reason, we proposed the high virulence Nc-Spain7 as a reference isolate, as it complies with all the above (48, 49, 53, 76, 77). Several bovine experimental infections using this isolate have been carried out in cattle at different times during gestation and under the same experimental design, which has allowed direct comparison of the results (53, 76–78). Specifically, intravenous inoculation of Nc-Spain7 tachyzoites showed a 100% of fetal death in a bovine model at early gestation (76, 77) and about 50% at mid-gestation (78). The intravenous route has been the most frequently used for experimental infections with *N. caninum*, as it simulates the hematogenous spread of the parasite after a primary infection or reactivation, likely allowing the parasite to reach the placenta quickly and in higher numbers. In contrast, parasites administered subcutaneously would target the regional lymph nodes first, and then would spread throughout the body, with lower numbers of parasites reaching the placenta. Intravenous inoculation of a high dose of tachyzoites (5 × 10^8^) of the Nc-1 isolate on day 70 of gestation induced 100% mortality, and this was reduced by half in subcutaneous inoculation (79), highlighting the relevance of using the first to mimic the most common route infection under natural conditions. The same research group compared the effect of inoculation of 10^7^ and 5 × 10^8^ Nc-1 tachyzoites by subcutaneous injection at day 140 of gestation. Both inocula induced similar outcomes, although the higher dose elicited earlier and more extensive lesions in the placenta (80). More recently, we have compared the effect of different doses of the Nc-Spain7 isolate, showing a dose-dependent effect on parasite loads in placenta and fetal brain, and in the median fetal survival times (81). A dose of 10^7^ tachyzoites intravenously administered at mid gestation was proposed to be adequate at modeling bovine pathogenesis because of its high abortion rate, placenta infection and parasite vertical transmission.

Similarly to what was described for *N. caninum*, experimental infections with *T. gondii* have been carried out under a large amount of different experimental designs (34). This prevents direct comparisons between studies and urges to the development of a normalized model of horizontal transmission. The reference type II isolates M1, M3, M4, and ME49 have been traditionally employed to perform experimental infections with *T. gondii* (82, 83). However, these isolates have been maintained by successive passages in cell culture and mice, in some cases for decades, and it is largely unknown if this have had any impact on their virulence traits as demonstrated for other type I isolates such as RH (84, 85). In fact, a recent study has demonstrated marked differences between the reference isolate ME49 and the recently obtained TgShSp1 isolate in terms of their *in vitro* behavior and virulence in mice, a model that has been historically used to classify all the *T. gondii* isolates into the three main clonal lineages, I, II, and III. Remarkably, susceptibility to neonatal mortality was higher in sheep than in mice, which corroborates that both the isolate and the animal model determine the outcome of infection, and as such, mice may not be a reliable indicator for disease severity (64).

Regarding the parameters that can be measured for studying parasite-placental interactions, these have been traditionally restricted to parasite detection and/or description of lesions in the placenta. Recent works have deepened in such interactions by investigating local immune responses in placentomes to elucidate its role in the clinical outcome of the disease, or in the control of parasite transmission (16, 53). However, current trends of research are moving forward the implementation of “omics” techniques (i.e., transcriptomics, proteomics, etc.) as they supply a global view of all processes altered during infection and potentially related to fetal death (58, 86).

##### Models for Bovine Neosporosis

Primoinfection models are the only experimental approach available to study bovine neosporosis, and they only mimic the exogenous transplacental route of transmission of *N. caninum*. In these models the time of gestation at which challenge is done determines the occurrence of fetal death or parasite vertical transmission, as well as the severity of pathological and immunological changes in the placenta (46).

Intravenous inoculation of heifers with tachyzoites of virulent isolates at the first term of gestation (65–70 days) is generally associated with a rapid fetal death due to the relative immunological immaturity of the fetus (46), and thus this model can be used to study pathological and immunological changes occurring in placentomes when fetal death occurs (Figure 3B). The results derived from these studies are quite homogeneous, with placentomes from aborted animals widely colonized by tachyzoites and displaying severe necrotic and extensive inflammatory lesions. With regard to cytokines expression at the maternal-fetal interface, an exacerbated pro-inflammatory response (particularly IFN-γ) and high expression of anti-inflammatory/regulatory cytokines (such as IL-10 or IL-4) were observed (76, 77, 87, 88). Moreover, a positive association between high number of T lymphocytes in placenta (CD3+, CD4+, CD8+, and γδ) and occurrence of abortion has been demonstrated by immunohistochemistry (IHC) (89). More recently, gene expression of the endosomal toll-like receptors (TLRs)-3, TLR-7, and TLR-8 by quantitative reverse transcription PCR (RT-qPCR) was described in the caruncle from cattle experimentally infected with *N. caninum* at day 70 of gestation and euthanized 1 month later (90), suggesting that the initial recognition of the parasite would occur in the maternal part of the placenta. Sequential euthanasia and sampling of cattle (Figure 3B) after intravenous challenge at early gestation, showed the presence of moderate to severe pathological changes and tachyzoites within areas of villous necrosis at 14 days post-infection (dpi) (79, 91). At 28 dpi, fetal death has occurred, and cotyledons were detached from caruncles, detecting *N. caninum* antigen in the cotyledons. When the immune response was studied by IHC, infiltration of CD4+ cells and NKp46+ cells was detected in the caruncles at 14 dpi, whereas infiltration of γδ TCR+ cells were observed from 28 dpi onwards (91). *In-situ* hybridization experiments demonstrated a more severe infiltration of IFN-γ expressing cells at 42 dpi, whereas the infiltrate of IL-4 expressing cells was scarce at 28 and 42 dpi, mainly in the caruncle (92). All these studies support the hypothesis that infection in early in gestation triggers a Th-1 type response in the placenta, which together with parasite replication and tissue destruction, may contribute to fetal death.

Experimental infections at mid gestation (110–140 days of gestation) can induce either foetopathy or birth of congenitally infected calves. It has been suggested that this model can be useful to understand why some dams abort and some do not. Here, the euthanasia of dams at a specific time of the experiment (Figure 3B) has been the experimental design most exploited so far. When the immune response was determined in placentomes from infected dams carrying live fetuses, Th1, Th2, and Treg cytokines were up-regulated, while TGF-β was down-regulated. This cytokine expression pattern could be beneficial to fetal survival, but could also promote transplacental transmission (93). When the immune response was determined in placentomes from aborted dams or dams carrying non-viable fetuses at the euthanasia, Th2 and Treg cytokines were down-regulated (71). Recently, cytokines expression pattern in Nc-Spain7 infected animals at mid-gestation, carrying non-viable fetuses and euthanized at 20 dpi, showed an up-regulation of IL-8, iNOS, and TNF-α, previously associated with placental inflammation, luteolysis and abortion (16). Other studies have described a reduced expression of SERPINA14 (linked to maternal immunosuppression during pregnancy) in aborting dams, and a negative relationship with IFN-γ expression in cotyledon samples (94). A recent work estimated the relative cell densities at the fetal-maternal interface from dams infected at day 110 of gestation, demonstrating lower proportions of bi- and mono-nucleate trophoblast cells after *N. caninum* infection at 42 dpi, and suggesting that this could be mediated by the Th1-type response of the placenta, promoting the destruction of fetal-genotyped cells (72). When infection dynamics were studied at mid gestation by serial euthanasia, the parasite was detected by PCR and IHC in the placenta as early as 10 dpi (53). Fetal death was detected from 20 dpi onwards (53, 80, 91, 95). Focal lesions and immune cell infiltration were observed in some placentomes at 14 and 28 dpi, and such lesions were resolved at later time points (80, 96). In non-aborting animals, there was no evidence of parasite antigen or infiltration of immune cells at 28, 42, and 56 dpi, suggesting that the parasite had not reached the placenta (91). In situ hybridization showed a scarce to mild infiltrate of IFN-γ, IL-4 expressing cells in the maternal caruncle at 14 and 28 dpi (92). Altogether these results suggest that infection at mid-gestation can be partially controlled by some dams, although the factors governing this remain unknown.

Experimental infection of cattle in the last third of gestation leads to the birth of healthy but congenitally infected calves. This model is useful to understand the basis of transplacental transmission and fetal survival. In placentomes obtained from animals euthanized 21 dpi, *N. caninum* was sporadically detected by PCR, very mild lesions with focal necrosis were found, and a modest expression of Th1 and Th2 cytokines was detected by RT-qPCR (87, 88). The presence of CD4+ and CD8+ cells was relatively low, and they were sparsely disseminated (88). Examination of placental tissues at different times post-infection evidenced the presence of parasites and focal necrotic lesions at 28 dpi, the infiltration of inflammatory cells at 42 dpi, and fibrosis at 56 dpi, which denoted lesion resolution. Animals euthanized at 14, 28, 42, and 56 dpi contained a scarce infiltrate of IFN-γ and IL-4 expressing cells, mainly in the caruncle (92). These findings indicate that infection at late gestation is less pathogenic at the maternal-fetal interface, which could partially explain the reduction on the severity of the clinical outcome after infection.

Pathological changes of *N. caninum*-challenged pregnant cattle at 70 days of gestation and previously immunized with live or inactivated experimental vaccines were also studied at the maternal-fetal interface. Heifers were culled at day 104 of gestation and placentomes were examined to evaluate lesions and local cellular immune responses using histopathology, IHC and RT-qPCR. Vaccination with live tachyzoites induced protective immunity, and thus, subsequent challenge only caused minimal inflammation, milder immune cell infiltration and a lower expression of cytokines. In contrast, animals vaccinated with inactivated vaccines displayed an up-regulation of Th1 and Th2 cytokines and a strong cellular immune response after challenge (97).

The influence of the *N. caninum* isolate on the pathological and immunological changes occurring in placentomes has been also studied in bovine experimental models. Inoculation of Nc-Spain1H and Nc-1 tachyzoites at early of gestation, resulted in death in three out of five fetuses from Nc-1 infected dams whereas that Nc-Spain1H did not have the ability to induce foetopathy. At the maternal-fetal interface, Nc-1 induced the most severe histopathological lesions and parasite DNA was also more frequently detected (52). In other studies using the same model, the high virulent isolate Nc-Spain7 induced fetal death earlier, severe placental damage and higher parasite load in caruncles than the moderate virulence Nc-1 (76) or Nc-Spain8 (77) isolates. In addition, higher IFN-γ expression was induced by Nc-Spain7, which could be detrimental for gestation, whereas a higher IL-10 upregulation was observed in Nc-Spain8 infected, which would have anti-inflammatory effects (77). More recently, we used a model of serial euthanasia for studying initial events of infection (10 and 20 dpi) at mid-gestation (53) to study the mechanisms that enable some isolates to be more effectively transmitted or cause fetal death. Infection with the low virulence Nc-Spain1H isolate triggered an early (10 dpi) innate immune recognition in the bovine placenta, characterized by upregulation of genes involved in pathogen recognition (e.g., TLR-2, TLR-3, TLR-8, TLR-9, and NOD2), inducing a solid Th1 response which were counterbalanced by a higher expression of anti-inflammatory and regulatory cytokines, minimizing placenta pathology. Conversely, signaling through pattern recognition receptors (PRRs, i.e., TLR-9, TLR-8) or cytokines (i.e., IL6, TGF-β1, IL-17A) was impaired at the placenta by the high-virulence isolate (Nc-Spain7), which may indicate the existence of an evasion mechanism that may favor an early multiplication and dissemination to the fetus. At the later stage (20 dpi), PRRs activation and a dramatically enhanced expression of the pro-inflammatory cytokines were observed. Altogether, this study demonstrated that the early activation of the innate immune response is crucial for the adequate control of *N. caninum* at the placenta, whereas an exacerbated pro-inflammatory immune response can lead to severe tissue damage, resulting in abortion. We went deeper in the study of the pathogenesis of the infection with these isolates of differing virulence using this model, by means of studying some components of ECM involved in maintaining placental homeostasis by IHC and RT-qPCR. Here, the low-virulence isolate promoted an ECM remodeling and tissue repair processes. However, in the placentome from animals infected by the highly virulent isolate, a profound alteration in ECM organization was observed, characterized by loss of fibronectin, vimentin and collagen in necrotic foci detected by IHC and downregulation of metalloproteases MMP-2, MMP-13, MMP-14, and their inhibitors. Recently, the lectin-binding pattern in the placentas of cows infected experimentally with *N. caninum* was studied by IHC techniques (98), suggesting the importance of the changes occurred in the ECM during *N. caninum* infection. In the last years, high-throughput technology has been used to generate relevant information on the immunological and cellular hallmarks of the host-parasite interaction at the placenta, aiming to facilitate further investigation on the key pathways influencing protection or abortion. Proteomic profiles comparison of the cotyledon and caruncle samples from previous experimental infections (53) has highlighted complement and coagulation routes, oxidation-reduction processes and ECM reorganization, among other biological processes, as key points in the pathogenic mechanism of *N. caninum* abortion (99). These mechanisms could play an important role and should be investigated in depth.

##### Models for Ovine Toxoplasmosis

Regardless the gestation term at which challenge was done (Figure 3), most of the infections documented so far have extended the length of the experiments until the occurrence of abortion, usually around 30 dpi. These studies described an extensive damage of the placenta caused by proliferating parasites, with lesions similar to those described in natural infections (multiple small foci of necrosis and non-purulent inflammation) (34, 44, 100). In these abortions, parasitic debris were observed in the maternal caruncle, whereas parasitic vacuoles were found in the trophoblasts of fetal cotyledon. This suggests that parasites are first recognized in the maternal part of the placenta where they stimulate the maternal immune response, and then spread to the fetus through infection of the fetal trophoblast villi, where they remain viable (69).

As opposed to the well-known “classical” abortion, early abortions occurring during the acute phase of the infection have been also described in some occasions, although they have not been taken into consideration until recent days (44, 64, 82, 101, 102). These works described early abortions in ewes infected at different stages of gestation, with different isolates (M4, ME49, and TgShSp1) and doses of inoculation. Although there was a correlation between the dose of infection and the rate of early abortions, a dose of just 50 sporulated oocysts was capable to cause a high rate of abortions in the acute phase of the disease and even 10 sporulated oocysts of the ME49 isolate caused early abortion in one animal (64, 82). In all cases, parasites were virtually not found in placental or fetal tissues, indicating that the pathogenic mechanism triggering early abortion is different from that observed in later abortions. Histopathological analyses revealed thrombosis and infarcts in the placentomes and ischemic lesions (periventricular leukomalacia) in the fetal brain, probably as a consequence of placental lesions (47, 82, 103). IHC analyses showed an increase of macrophages in caruncular septa, which could indicate that early abortions are immune-mediated. Nevertheless, more studies are necessary to corroborate this hypothesis and to find out the origin of the placental thrombosis observed. In this sense, experimental models of serial euthanasia at early stages post-infection along with high-throughput approaches to dissect host-parasite interactions could prove useful.

Recent attempts to reproduce the classical late abortions described in natural infections have failed, and early abortions have turned into the predominant clinical presentation of experimental toxoplasmosis despite they have been virtually neglected from the literature until now. Specifically, infection with 2,000 and 500 sporulated oocysts resulted in almost 100% of early abortions. This rate was significantly reduced with lower challenge doses but, surprisingly, this reduction was not counteracted by an increase on the rates of late abortions, and infections with 50 sporulated oocysts or less resulted in the birth of healthy lambs, stillbirths and very few late abortions (64, 82). Therefore, the development of a model able to mimic the classical late abortions described in natural infections is an urgent need. Factors such as breed, previous immunization or even individual susceptibility should be considered in new experiments.

A number of studies aimed to investigate *T. gondii* infection dynamics through models of serial euthanasia (44, 47). However, these works failed to describe the mechanisms underlying early abortions (infected ewes were euthanized from 10 dpi onwards once most of those abortions had occurred) and focused on the late ones. Although in very few animals, *T. gondii* was detected in placenta as early as 10–12 dpi; in addition, percentages of detection and parasite loads were higher as the infection progressed. Development of lesions was observed as soon as 10 dpi, but only in ewes subcutaneously inoculated with tissue cyst at 60 or 90 days of gestation (44), an uncommon way to acquire the infection in the nature. These lesions were characterized by several foci of necrosis that became larger as the infection progressed. In ewes orally infected with sporulated oocysts, typical lesions were found in placenta from 26 dpi, always associated to the presence of the parasite, and were characterized by necrotic foci in the caruncular part and infiltration of macrophages and T lymphocytes within the fetal chorionic villi (69, 82).

Similarly to bovine neosporosis, the time of gestation at which infection occurs also influences lesion severity, parasite distribution and expression of cytokines at the placenta, but very few studies have addressed these matters. As early as 1986, Buxton et al., analyzed the placental lesions developed in pregnant ewes after subcutaneously inoculation of 200 tissue cysts of the M1 isolate at 40, 60, and 90 days of gestation and at the infection outcome (44). This study described the occurrence of necrotic lesions in caruncular septa, which were more severe in ewes infected at mid-gestation and in those where fetuses were dead. More recently, similar studies were done using pregnant ewes orally inoculated at early- (40 days of gestation), mid- (90 days of gestation), and late-gestation (120 days of gestation) with sporulated oocysts of M4 isolate (17, 47, 69, 100). Infection at mid-gestation also resulted in the most severe lesions and highest parasite burdens, both in the maternal and fetal placenta. However, ewes infected at late-gestation showed the earliest development of lesions and colonization of the placenta by the parasite (47). Castaño et al. hypothesized that these variations could be due to differences in the immune modulation along the pregnancy. While pro-inflammatory cytokines (IFN-γ and TNF-α) were equally increased during the three terms of gestation, IL-4 was mainly increased at the first and second terms, and IL-10 at the last term of gestation. Besides, a regulation of IL-12 expression was not observed at any term (17). This imbalance on the Th1 and Th2 responses could play a key role on the pathogenesis of toxoplasmosis, but this issue remains controversial among the scientific community.

### *In vitro* Models

Over the last years, a number of *in vitro* human placenta models have been developed and widely used for studies in different animal and human parasites (104), and similar efforts have been performed to obtain both primary and established cell lines from the bovine and ovine placenta (Table 1).

**Table 1:** Primary and established cell lines isolated from the ruminant placenta.

**Specie**	**Origin**	**Cell type**	**Cell line**	**References**
BOVINE	Blastocyst	Trophoblast cells	BE-13	(105)
			CT-1	(106)
			-	(107)
			BT-1	(108)
			-	(109)
			BT-(A-L)	(110)
			-	(111)
			-	(112)
	Amnion	Trophoblast cells	biTBCs	(113)
	Cotyledon	Trophoblast cells	-	(114)
			-	(115)
			F3	(116)
			Line A, Line B	(117)
	Caruncle	Caruncular epithelial cells	-	(118)
			BCEC-1	(119)
OVINE	Cotyledon	Binucleate cells (BNC)	-	(120)
			-	(121)
		Trophoblast cells	-	(122)
			-	(123)
			Otr1, OtrF	(124)
			-	(125)
			oTr	(126)
		Immortalized trophoblastic cells	AH1	(127)
			hTERT-STCs	(128)

Despite the available number of such cell lines is considerable, most of them are not suitable for conducting studies on host-parasite interactions. This is because they are usually contaminated with *Mycoplasma* spp. and the bovine viral diarrhea virus (BVDV). Infection with BVDV is common in bovines and not routinely tested, but its presence in cell culture has demonstrated to alter host-pathogen interactions in an *in vitro* model of *Besnoitia besnoiti* infection (129). Considering this, and to ensure results reproducibility and reliability, selection of appropriate cell lines is paramount. In this section we discuss the application of available and suitable *in vitro* systems for the study of the pathogenesis of neosporosis and toxoplasmosis, including their advantages and disadvantages and the emerging opportunities that arise from their use.

#### Bovine Placenta Cell Lines

Two different cell lines, BCEC-1 and F3, both derived from bovine placentae and representing the main maternal and fetal cells that constitute the placental barrier have been successfully used to perform *in vitro* infections with *N. caninum*. The caruncular cell line BCEC-1 (bovine caruncular epithelial cell line-1) was isolated from a pregnant cow at 4 months of gestation, proved to have maternal origin, and maintained cell morphology, protein expression profile and barrier integrity for at least 32 passages (118, 119). A few years later, the bovine trophoblast cell line (F3) was isolated from cotyledons of pregnant cows at 5 months of gestation. This cell line was proved to have fetal origin, displayed epithelial morphology, supported the development of BNCs in small numbers through all passages, and maintained these features beyond 45 passages (116). The use of both cell lines has helped to unravel several aspects of host-parasite interactions in neosporosis.

##### Study of the Lytic Cycle in *N. caninum*

During the course of a natural infection, parasite survival and propagation heavily rely on successful invasion and proliferation within receptive host cells as part of the parasite's lytic cycle (130). Thus, the study of such processes in target cells appears as an ideal tool to study host-parasite interactions at the placental barrier. Our previous work demonstrated that *N. caninum* actively replicates in caruncular and trophoblast cells, confirming the high tropism of the parasite for the placenta observed *in vivo* (56). However, we found interesting differences in the behavior of *N. caninum* between both cell lines. Immunofluorescence assays showed that parasites displayed a lower adhesion capacity to caruncular cells, and consequently, the infection rates and percentages of cells with multi-infection were lower in BCEC-1 cells. In addition, parasite proliferation capacities, measured by RT-qPCR, were also lower in those cells. These results reflected that susceptibility to infection is higher in the fetal part of the placenta, which is widely supported by previous results obtained from experimental bovine infections (16, 76, 77). In view of these, we proposed that the maternal part of the placenta acts as a barrier for parasite infection, reducing its adhesion and subsequent intracellular multiplication.

**Figure 4:**
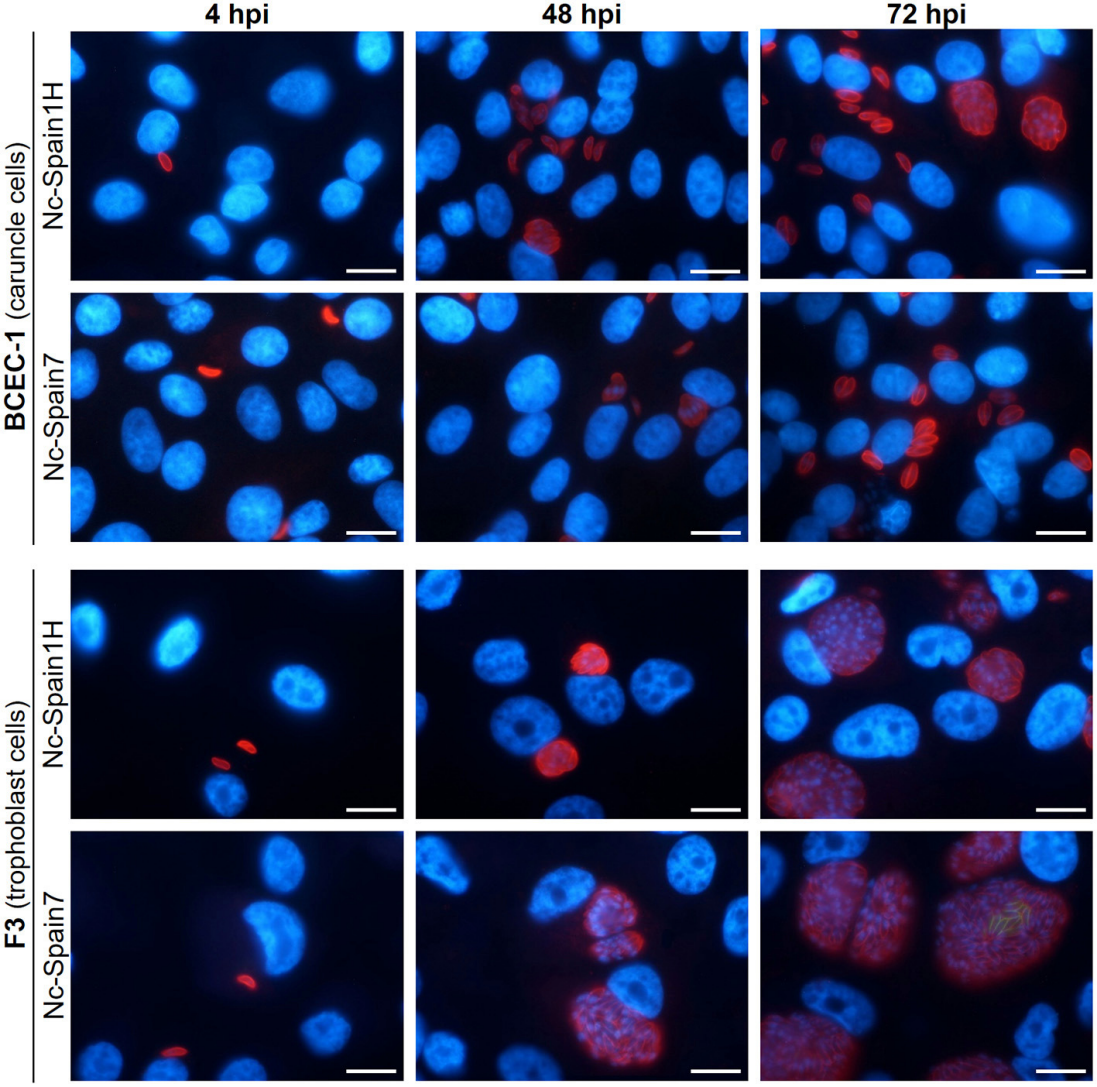
Tracing the lytic cycle of high- (Nc-Spain7) and low-virulence (Nc-Spain1H) isolates of *N. caninum* in BCEC-1 and F3 cells at 4, 48, and 72 h post-infection (hpi) through immunofluorescence assays. Host nuclei were stained in blue, while *N. caninum* tachyzoites were stained in red. Note the obvious differences in the parasitophorous vacuole size between BCEC-1 and F3 cells infected with any of the isolates, which is due to an early egression of the parasites in the first. While no differences were observed in parasitophorous vacuole size between BCEC-1 cells infected with Nc-Spain1H or Nc-Spain7 tachyzoites, remarkable differences were evidenced in F3 cells, with bigger vacuoles in Nc-Spain7 infected cells compared to those infected with Nc-Spain1H. Note the beginning of egress in F3 cells infected with the Nc-Spain7 isolate at 72 hpi (stained in green). Scale-bars: 10 μm.

On the other hand, pathogenesis of *N. caninum* infection is heavily influenced by the virulence of the isolate employed. We hypothesized that differences in lytic cycle progression among different isolates could explain the differences described *in vivo* using a bovine model (52, 76, 77). In an attempt to address this possibility, the lytic cycle of the high-virulence isolate Nc-Spain7 and the low-virulence isolate Nc-Spain1H was compared using the BCEC-1 and F3 cell lines (56). Differences between isolates were remarkable in trophoblast cells, where Nc-Spain7 displayed higher invasion and proliferation rates than Nc-Spain1H. By contrast, the behavior of both isolates was very similar in caruncular epithelial cells (Figure 4). This higher proliferation capacity in trophoblast cells could explain why in heifers experimentally infected with the Nc-Spain7 isolate show higher parasite burdens in fetal tissues, and consequently, more severe clinical signs and histopathological lesions, resulting in higher ratios of fetal death and abortion.

##### Study of the Host-Cell Modulation by *N. caninum* Infection

It has been widely demonstrated that parasites and many other pathogens can manipulate host-cell machinery for their own benefit in order to perpetrate the infection by scavenging nutrients from the host, or even by inhibiting apoptosis or host immune responses. In addition, it has been shown that the pathogenesis of bovine neosporosis is not only related to parasite multiplication itself, but also to the host immune response triggered following infection (131). Therefore, the study of the host-cell modulation upon *N. caninum* infection could contribute to deepen on the knowledge of the disease.

The application of classical techniques such as RT-qPCR and commercial bovine cytokines ELISA tests to this *in vitro* infection model have proven to be very useful to study the response triggered by *N. caninum* in bovine target cells. This was revealed by studying the expression levels of cytokines, chemokines and other immune-related elements in F3 and BCEC-1 cell cultures infected with high- (Nc-Spain7) and low-virulence (Nc-Spain1H) isolates at early (4 h) and later (24 h) stages of infection (57). Similarly to what has been proven using *in vivo* models, this work described the development of a typical pro-inflammatory Th1 response by the bovine host against the parasite. Surprisingly, and despite the different phenotypes observed between BCEC-1 and F3 cells in terms of parasite adhesion, infection rates, and proliferation capacities, few differences were found in their responses against the infection. The main difference was the up-regulation of TLR2 in infected BCEC-1 cells, not found in F3 cells. This phenomenon could be in part responsible of the resistant phenotype found in caruncular cells, as TLR activation is crucial for initiating the immune responses against intracellular parasites such as *N. caninum*. However, the different TLR-2 activation did not influence the expression of TNF-α, IL-8, and IL-6, usually triggered by transcription factors also activated by this receptor. Regardless of TLR-2 expression levels, the pro-inflammatory cytokines TNF-α and IL-8 were in general up-regulated upon infection in both cell lines. Surprisingly, IL-6 expression levels were diminished in infected cultures of BCEC-1 and F3 cells. These results deserve further investigation, since a marked IL-6 up-regulation has been described upon *N. caninum* infection *in vivo* (16, 93, 132). On the other hand, TGF-β1 was found down-regulated in BCEC-1 and F3 cells. It is postulated that this factor might contribute to fetal death during infections, as it neutralizes the pro-inflammatory responses induced by Th1-type cytokines whose activity is detrimental for the gestation. When immune modulation was individually compared after infection with high- or low-virulence isolates, only differences on TLR-2 and TNF-α expression levels were found, with the Nc-Spain1H isolate inducing higher expression of TLR-2 and TNF-α than the Nc-Spain7. This could be translated into a better control of parasite proliferation by the cells infected with the Nc-Spain1H isolate, or the opposite in those infected with the Nc-Spain7 one (57).

Despite this model has demonstrated to be useful to unravel some aspects of *N. caninum* pathogenesis, its use dismisses the close interaction of caruncular and trophoblast cells with other immune cells present in the placenta. This is clearly reflected by the lack of detection or down-regulation of cytokines such as IL-17, IFN-γ, IL-4, IL-10, and IL-12, whose relevance in the response against *N. caninum* in bovine models has been long-established (16, 93, 132). These limitations could potentially be overcome by exogenous stimulation of F3 and BCEC-1 cultures with cytokines such as IFN-γ present at the placenta, or even by co-culturing these cell lines with other relevant bovine immune cells such as macrophages. Additional alternatives such as the use of placental explants are discussed below.

Although the use of classical methods has provided relevant information on the expression and abundance of many genes of interest upon infection with *N. caninum*, they fail to provide a comprehensive view of the global changes that infection might trigger on host target cells. In this regard, recent advances in high-throughput technologies have provided an opportunity to perform a global analysis of potential changes related to infection using trophoblast cells (58, 86). Infections of F3 cells with high- (Nc-Spain7) and low-virulence (Nc-Spain1H) isolates demonstrated that the parasite modulates processes such as the ECM reorganization, cholesterol biosynthesis and the transcription factor AP-1 network after just 8 hpi. Specific isolate-modulated processes were not identified, although infection with the low-virulence isolate exerted a higher modulation of the host cell (207 differentially expressed genes) compared to the high-virulence one (126 differentially expressed genes) (58). Subsequently, and in order to depict the infection dynamics throughout the tachyzoite lytic cycle, the proteome of *N. caninum*-infected F3 cells was studied by LC/MS-MS during early infection (8 hpi), parasite multiplication (36 hpi), and egress (56 hpi). Proteome changes revealed protein synthesis, protein turnover, and metabolism as the main host pathways disturbed by infection. Similar findings have been reported for other parasites, as manipulation of host metabolic events determines infection establishment and the initiation of host immune responses (58, 133–135). As observed in the transcriptomic study, infection with the low-virulence isolate Nc-Spain1H exerted a higher perturbation on F3 cell proteome with the exception of those profiles enriched in mitochondrial proteins, which were equally altered by high- and low-virulence isolates. This reflects the importance to govern this organelle during infection, which has been broadly demonstrated not only for *N. caninum*, but also for *T. gondii* (136–139). Nevertheless, the levels of perturbation of the host cell depending on the isolate's virulence deserves further investigation, as this could be a strategy to achieve a more successful transmission (higher perturbation by low-virulence isolates), or to avoid detection by the host's immune response (lower perturbation by high-virulence isolates). Unfortunately, there is a lack of similar studies performed in bovine caruncular cells, where the parasite has shown an uncommon behavior not described in other cells lines (49, 140). This could provide valuable information about the interactions between *N. caninum* and the maternal side of the placenta.

#### Ovine Placenta Cell Lines

Sheep are susceptible to infection by both *N. caninum* and *T. gondii* (100, 141). Even considering their limitations, the use of *in vitro* models of ovine placenta would provide useful information that could be extrapolated to the natural conditions of infection by both parasites. However, the number of studies that have adopted this model is strikingly scarce to date. Back in 2006, the AH-1 cell line was originated from the ovine placenta to perform *in vitro* infections using *N. caninum* tachyzoites (127). This cell line was developed from fetal cotelydon cells obtained from a near-term pregnant Suffolk ewe by transformation with the SV40 large T antigen. AH-1 cells displayed a morphology consistent with that observed in freshly obtained trophoblasts, and were able to produce IFN-τ, a cytokine nearly unique to the ruminant placenta. Haldorson et al. also demonstrated that antibodies directed against the surface protein NcSRS2 were able to inhibit *N. caninum* cell attachment and invasion *in vitro*. Nevertheless, to our knowledge this is the only study where the AH-1 cell line has been used to deepen on the pathogenesis of *N. caninum*. This contrasts with other works focused on the abortifacient agents *C. abortus* and *Waddlia chondrophila*, which are able to infect and grow in this cell line, which in return triggers a typical pro-inflammatory response (142, 143), demonstrating its great potential to study not only *N. caninum*, but also *T. gondii*.

Regarding *T. gondii*, and considering its zoonotic nature, many *in vitro* studies have been performed using human placenta cell lines. Among them, the BeWo cell line (human choriocarcinoma cells) has been the most widely used for studies on the parasite's pathogenic mechanisms, host immune response or drug efficacy upon *T. gondii* infections (144–146). Since abortion is the main consequence of *T. gondii* infection both in humans and ovine, studies based on BeWo cells could guide future research in ovine target cells. In this sense, the use of the AH-1 line could be a good starting point, but further efforts should be made to obtain an established cell line representing the maternal side of the ovine placenta. We have recently characterized the lytic cycle of six *T. gondii* isolates newly obtained from ovine tissues on infected AH-1 cells, and we can certainly corroborate their usefulness for this end (data not published).

### *Ex vivo* Models

The term explant refers to a piece of a living tissue or organ that once removed from an animal and transferred to artificial culture conditions is able to maintain its functions for a short period of time, thus allowing to perform controlled experiments on it. During the last 80 years the use of placental explants for research in human medicine has been extensively reported, and has enabled the study of the placental physiology and certain pathologic conditions such as preeclampsia and gestational diabetes (147). In fact, it has been demonstrated that these explants are able to maintain their degree of cell differentiation and secrete hormones and cytokines under artificial culture conditions (148). This contrasts with the scarce works carried out with ruminant placental explants, which reflects the difficulties in obtaining placentas suitable to tissue culture from those farms where births take place.

#### Practical Applications of Placental Explants

To date, the use of placental explants has not been reported to study the effect of *N. caninum* and *T. gondii* in ruminants. However, the literature available supports their employment as a valuable approach capable of dissecting the pathogenic mechanisms and host responses triggered upon infection, thus overcoming the limitations of caruncular or trophoblast cells-based *in vitro* cultures. This is further backed up by several studies where human placental explants have been infected *ex vivo* (Table 2).

**Table 2:** Compilation of studies where human placental explants have been employed to study host-parasite interactions *ex vivo*.

**Parasite used for infection**	**Main findings in human placental explants**	**References**
*T. gondii*	Infection induces the secretion of the macrophage migration inhibitory factor (MIF), up-regulates ICAM-1 expression, and increases monocyte adhesiveness to trophoblast	(149)
	Parasite burdens are determined by the expression of MIF receptors and its secretion, which varies with the trimester of gestation	(150)
	The extravillous trophoblasts (fetal) are preferentially colonized over the syncytiotrophoblast (maternal), which is linked to an up-regulation of the chemoattractant CCL22	(151, 152)
	Parasite proliferation relies on iron acquirement from the placenta	(153)
	Azithromycin, enrofloxacin or toltrazuril modulate MIF production by explants and reduce parasite replication	(145, 154, 155)
	Infection inhibits the non-canonical NF-κB pathway through TLR-4 and TLR-9 related mechanisms, resulting in higher parasite burdens	(156, 157)
*T. cruzi*	The syncytiotrophoblast (maternal placenta) limits infection through the production of nitric oxide	(158–160)
	Infection induces trophoblast differentiation, tissue disorganization, destruction of chorionic villi and apoptosis	(160–164)
	TLR-2 activation and the NF-κB pathway are key to control infection	(156, 157, 165)
*P. falciparum*	Infection alters the Th2 bias of maternal immunity and elicits a Th1 response	(166, 167)

Very few studies have employed cow explants to study specific aspects of the placental physiology (168). In contrast, recent endeavors to obtain inexpensive tools to study the consequences of xenobiotics exposure on the maternal-fetal interface have resulted in the development of an *ex vivo* model based on sections of cow placentomes (169). In fact, this model has been applied to describe the effect of environmental endocrine disruptors on the bovine placenta (170). Of more interest for this review is the use of bovine placental explants to extend what is known about the pathogenesis of *B. abortus*-induced placentitis and abortion in cattle. The experiments carried out with extraplacentomal chorioallantoic explants from early and late gestational stages showed that bacterial replication was significantly higher in last term tissues (171). Further studies demonstrated that infection with *B. abortus* suppresses the pro-inflammatory innate immune response triggered by trophoblastic cells (172, 173). Similar approaches have been followed to study the pathogenesis of *Listeria monocytogenes* and *Listeria ivanovii*, also responsible of abortion and reproductive failure in cattle. Both species displayed similar levels of invasion and intracellular multiplication of chorioallantoic explants without inducing cell lysis. Oppositely to *B. abortus*, no significant changes on host pro-inflammatory responses were observed after infection (174).

Regarding ovine *ex vivo* models, the number of works where placental explants have been employed is limited, and most of them have focused on the study of the placental physiology (175). Nevertheless, it is worth to mention a recent work in which the effect of *ex vivo* infection with *Trypanosoma cruzi* and *T. gondii* was compared between three different placental barriers: human, canine and ovine. This study demonstrated that the parasite burden seemed to be independent of the placenta's complexity. Besides, this burden was always higher in those explants infected with *T. gondii*, where the tissue damage tended to be more severe (176).

In contrast to ruminant placental explants, human extravillous explants have been extensively used during the last decades to study many aspects of placental physiology and pathology. This has been favored by the relative ease with which these tissues can be obtained during scheduled cesarean sections or pregnancy terminations. Parasites such as *T. gondii, T. cruzi*-the etiological agent of Chagas disease-, and *Plasmodium falciparum*-responsible of placental malaria- are a significant cause of placentitis, premature labor, miscarriage and low birth weight in humans. The major findings of these works are summarized in Table 2 in an effort to serve as a route map for future research that could be applied in the field of neosporosis and toxoplasmosis in ruminants.

In summary, the development of *ex vivo* models for the bovine and ovine placenta opens up new prospects to study in depth the intricated relationship between apicomplexan parasites and their hosts during pregnancy, and this will help to understand the consequences of such infections to the developing fetus.

#### Placental Explants: Pros and Cons

The employment of an *ex vivo* approach to model placental alterations features diverse advantages and drawbacks that have been extensively reviewed earlier (177). The explant-based models benefit from maintaining the tissue architecture and the extracellular matrix between all the cell-types that shape the placenta, thus reproducing *in vivo* physiologic conditions better than *in vitro* systems (178). Cells can keep the physical contact with the basal lamina, allowing the reception of paracrine signals from the underlying layers, and the presence of immune cells enables the study of unique immunomodulation processes that could be unnoticeable through *in vivo* approaches and undetectable *in vitro*. This makes the explants an excellent tool for studying parasitic infections and local antiparasitic mechanisms as demonstrated previously (179). The use of placental explants also offers several advantages including an increased number of variables to be tested in a single experiment, shorter experimental times, and reduced individual variability between experiments. In addition, explants could be isolated from placentas at different terms to investigate the impact of gestational age in a given parameter (148, 177). From an ethical point of view, the utilization of *ex vivo* models meets the principle of the “Three Rs” as the use of few animals would provide enough tissue to test a vast amount of experimental conditions.

Despite all of the above-mentioned, the utilization of placental *ex vivo* approaches is not exempt of some disadvantages. One of its major drawbacks is the short lifespan of explants, as they can only be used right after tissue collection, thus impeding to be tested later. This approach is also constrained by the limited availability of tissues suitable for explant cultures. Abattoirs are an ideal source to obtain placentas, but considering that breeding costs are relatively high, it is likely that pregnant females slaughtered there are suffering from underlying health conditions that would prevent their inclusion as tissue donors. Similarly to human studies, cesarean sections could constitute a practical way to obtain ruminant placental explants. Nevertheless, these surgeries require an experienced surgeon and are always an emergency procedure. As such, it is difficult to predict when and where the tissues will be available, and if blood flow has been compromised on them. On the other hand, the use of naturally expelled placentas from natural births is not even considered for this purpose, as this is a long process that in most cases results in a rapid tissue autolysis. In this context, placentomes obtention from pregnant females with known sanitary status and exclusively euthanized for this purpose emerges as an ideal alternative to overcome the mentioned drawbacks.

Tissue explants might be subjected to cryopreservation methods that would allow their isolation in a standardized way, thus reducing variability between experiments, and their usage under an on-demand basis (104). Availability of tissue biobanks from healthy donors would enable homogenous experimental designs and accelerate the obtention of reliable conclusions. The field of tissue cryopreservation has been widely expanded in human reproduction, where protocols for long-term conservation of oocytes, sperm, embryos, and even ovary slices have been long established (60). Nevertheless, cryopreservation of entire tissues is not without challenges due to their complex cell diversity and structure.

Despite that, various successful methods are now available to freeze human and canine placentas. These methods are based on controlled cooling rates and the use of cryoprotectants (60, 180). The employment of such protocols has demonstrated that cryopreservation does not significantly affect the viability of cells, their functional activity, proliferation, differentiation capacity, or their mitochondrial respiration, and only causes some damage to the mesenchyme (60, 180, 181). Consequently, the development of cryopreservation protocols for placental explants in ruminant species and their subsequent use for the study of diseases that cause reproductive failure seems feasible.

## Concluding Remarks

Most of our current understanding on the pathogenesis of neosporosis and toxoplasmosis has emerged from the use of experimental models, but there are still important gaps on the placental host-parasite interactions that must be addressed to promote the development of effective control measures against both diseases in the future. *In vivo* models are indisputably the best available tools to secure reliable information about the mechanisms responsible of reproductive failure, but a number of factors such as the parasite isolate, challenge dose, route of inoculation, term of gestation, and the time of sampling have a substantial impact on the infection outcomes. Special efforts should be made to develop standardized models for each disease, or at least follow thoroughly previous experimental designs available in the literature, which will permit direct comparisons between works. However, the use of *in vivo* models incurs higher costs, longer experimental periods, and ethical constraints, making necessary the use of alternative models. *In vitro* models have proven useful to describe the behavior of *N. caninum* after infection of the main cell populations present in the bovine fetal-maternal interface, but much work remains to be done for *T. gondii*. Selection of appropriate cell lines is paramount to ensure reliable and reproducible results, but once obtained, they offer a cost-effective and animal-free source of experimentation material that can be easily exploited. Nevertheless, the use of *in vitro* approaches neglects the close interaction of trophoblast and caruncle cells with the immune populations locally present in the placenta. This problem could be solved by exogenous cytokine stimulation of responsive cells, or even with relevant immune cells, and should be explored in the future. Additional alternatives such as placental explants have the potential to overcome the limitations of the *in vitro* models, as they maintain the tissue architecture and preserve immune populations present in the placenta, but considering their short lifespan and limited availability, their application to study host-parasite interactions in the placenta will be limited until suitable cryopreservation methods have been developed. In all cases, complementation of classic techniques such as PCR and histology with more advanced techniques such as RT-q-PCR, IHC, transcriptomics, and proteomics, have the potential to offer a global view of all processes that result significantly altered during infection with both parasites.

## Author Contributions

All authors listed have made a substantial, direct and intellectual contribution to the work, and approved it for publication.

## Conflict of Interest

The authors declare that the research was conducted in the absence of any commercial or financial relationships that could be construed as a potential conflict of interest.
